# Rapid testing of antibiotic residues to increase food safety awareness of animal origin

**DOI:** 10.14202/vetworld.2024.1177-1183

**Published:** 2024-05-28

**Authors:** Dyah Ayu Widiasih, Reza Putra Pratama, Yatri Drastini, Khrisdiana Putri, Laila Nur Fatimah, Soedarmanto Indarjulianto

**Affiliations:** 1 Department of Veterinary Public Health, Faculty of Veterinary Medicine, Universitas Gadjah Mada, Yogyakarta, Indonesia; 2 Department of Agriculture, Horticulture and Livestock, Jambi, Indonesia; 3 Department of Internal Medicine, Faculty of Veterinary Medicine, Universitas Gadjah Mada, Yogyakarta, Indonesia

**Keywords:** antibiotic residual level, antimicrobial resistance, food animal origin, food safety, rapid screening test

## Abstract

**Background and Aim:**

Antibiotics are used to improve growth, reduce disease, and decrease mortality in animals grown for food. The government regulates and prohibits the use of antibiotics, in particular, the use of antibiotic growth promoter (AGP) in livestock; however, it is not yet known whether the use of antibiotics is in accordance with regulations so that there are no antibiotic residues in food of animal origin. To ensure food safety of animal origin and to raise awareness of food safety, it is necessary to detect antibiotic residues in fish, eggs, and chicken meat from Yogyakarta Special Province through monitoring and monitoring. To ensure food safety and regulatory compliance in food samples, antibiotic residue screening techniques are essential. A number of methods, such as time-consuming and costly chromatographic and spectroscopic methods, have been developed for the detection of antibiotic residues in food samples; however, not all laboratories have these facilities. Therefore, a rapid diagnosis of food of animal origin is required. The purpose of this study was to rapidly test antibiotic residues by using Premi^®^test kits (R-Biopharm AG, Germany) to increase awareness of food safety of animal origin.

**Materials and Methods:**

We tested 345 animal-based food samples from traditional markets, supermarkets, and central markets in five districts of Yogyakarta Special Province for antibiotic residues using rapid test kits and observation questionnaires to identify risk factors.

**Results:**

The presence of antibiotic residues in food-animal origin samples from the Yogyakarta region had an antibiotic residue level of 9.28% (32/345), consisting of fish samples 11.3% (18/97), eggs 15.65% (1/114), and chicken meat samples 0.87% (13/102). The highest percentage of samples positive for residual antibiotics was 21.9% (7/32) from supermarket meat samples. The highest amounts of antibiotic residues were found in fish samples collected from Sleman Regency, up to 25% (8/32), whereas in supermarket fish samples, there were as high as 18.8% (6/32).

**Conclusion:**

Antibiotic residues in animal-based food can be attributed to various factors, including product source, transportation conditions, and environmental conditions. The widespread distribution of antibiotic residues in fish comes from environmental conditions during maintenance, distribution, and retailing. Monitoring antibiotic residue prevalence in food-animal origins, particularly chicken meat, eggs, and fish, is crucial for improving animal food quality and safety.

## Introduction

Antibiotics are active ingredients used to treat bacterial diseases in humans and animals. Infectious diseases have been solved by the discovery of antibiotics, which are recognized as the cornerstones of modern medicine [1]. Animal diseases, such as mastitis, arthritis, respiratory distress, gastrointestinal infections, and other bacterial infections, are all treatable and preventable with antibiotics [2]. Antibiotics are one of the most important aspects of veterinary medicine in animal feed and food production [2, 3]. The use of antibiotics on animals raised for food has significantly improved animal health by encouraging the development of livestock, reducing the incidence of disease and death, and generally improving animal health. Antibiotics also significantly increase agricultural productivity [4]. Antibiotic growth promoters (AGPs) are antibiotics used in animal feed to promote growth and prevent disease.

Since 1940s, the use of AGP has been a common practice in the animal industry [5]. Concerns about the development of antimicrobial resistance (AMR) have led to restrictions on the use of AGP in many countries [6]. The Indonesian government has also regulated and banned the use of antibiotics, particularly AGP, in livestock [7, 8]. However, the use of antibiotics is often misused and food safety concerns are raised [9]. Food safety concerns are significantly influenced by the general public’s ignorance of AMR, which is caused by the buildup of antibiotic residues in food of animal origin and their ignorance of the adverse health consequences of AMR [10, 11]. Antibiotic residue screening techniques are therefore essential for ensuring food safety and compliance with food safety regulations. Various approaches, such as time-consuming and expensive chromatographic and spectroscopic procedures, have been developed for the identification of antibiotic residues in food samples [12]. In Yogyakarta, high-performance liquid chromatography (HPLC) has been used to identify tetracycline residues in tilapia liver from traditional markets [13], whereas bioassay methods have been used to identify antibiotic residues in chicken meat and chicken eggs in Yogyakarta City [14]. A previous study by Maulana *et al*. [15] indicated that milk, a food item of animal origin in Sleman Regency, Yogyakarta, contained antibiotic residues and even included *Escherichia coli* bacteria that produce extended-spectrum beta-lactamases-producing *E. coli*. These bacteria may cause resistance to antimicrobials in food of animal origin. However, rapid testing kits for the detection of antibiotics of animal origin have not been widely reported or disseminated to the public. In view of the lack of information on the origin of safe and useful animal foods, research on rapid tests of antibiotic residues to raise awareness of food safety is crucial for protecting public health and ensuring food safety.

The aim of this research was to rapidly test antibiotic residues by using Premi^®^test kits in animal-derived food products, raise food safety awareness, and develop public education materials on avoiding antibiotic residues, healthy food choices, and sparing antibiotic use.

## Materials and Methods

### Ethical approval

This study did not involve living creatures; therefore, it does not require ethical approval.

### Study period and location

**Figure-1:**
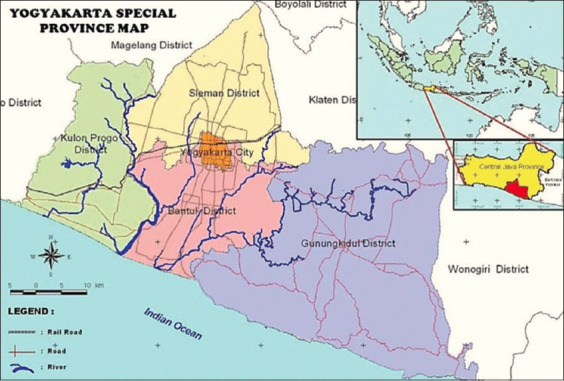
Sampling area in the Yogyakarta Special Province [16].

The study was conducted from August to December 2021. This study was conducted in five areas: Bantul District, Gunung Kidul District, Yogyakarta City, Kulon Progo District, and Sleman District in the Yogyakarta Special Province Area (Figure-1) [16].

### Sampling size

Purposive sampling was employed in this study to examine animal-derived foods offered in traditional markets and supermarkets in Yogyakarta special province. Using the formula n = 4PQ/L^2^ [17], we calculated the number of samples and obtained 345 food samples of animal origin (fish, eggs, and chicken meat).

### Test procedure

Fish, egg, and chicken samples were selected on the basis of the consumption trends of people in Yogyakarta Special Province. Premi^®^test kits (R-Biopharm AG, Germany) were used to analyze antibiotic residues in food samples of animal origin. Vendors receive surveys and observations to assess the risk factors associated with antibiotic residues in animal products. The collected data were subjected to descriptive analysis.

## Results

**Figure-2:**
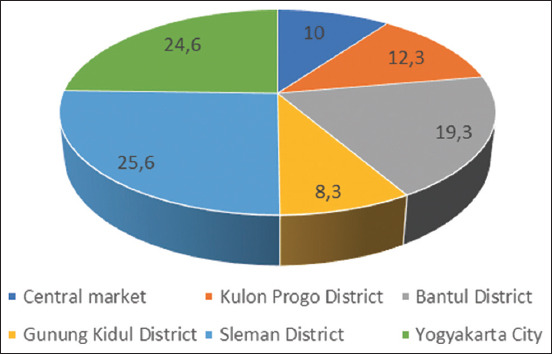
Percentage of sampling area in Yogyakarta Special Province.

Sleman District (25.6%), followed by Yogyakarta City (24.6%), Bantul District (19.3%), Kulon Progo District (12.3%), and Central Market (10%), had the highest percentage of food-animal origin samples (Figure-2).

Furthermore, Table-1 shows the positive percentage of antibiotic residues in samples originating from food animals.

**Table 1:** Results of food-animal-origin samples antibiotic residue screening in Yogyakarta Special Province.

Sample Type	Number of samples	Positive (+)	Negative (-)	Positive percentage
Chicken	115	13	102	11.30
Fish	115	18	97	15.65
Egg	115	1	114	0.87
Total	345	32	313	9.28

Antibiotic residues were found in 11.3% (13/115) of chicken meat samples, 15.65% (18/115) of fish samples, and 0.87% (1/114) of egg samples according to the results of the rapid antibiotic residue test. Overall, based on the total number of samples tested, the residual antibiotic content was 9.28% (32/345) in food samples of animal origin in the Yogyakarta Special province area.

From the data in Table-2, samples of chicken meat from supermarkets had the highest percentage of positive results (21.9% [7/32]) of all positive samples. The concentration of antibiotic residues in fish was found to be as high as 25% (8/32) in Sleman Regency samples and as high as 18.8% (6/32) in supermarket samples.

**Table 2:** Positive sample data based on food animal origin samples from the Yogyakarta Special Province.

Food animal origin samples	Regency/City							

	Bantul	Sleman	Yogyakarta City	Gunung Kidul	Kulon Progo	Supermarket	Central Market	Total
Chicken	0	1	0	2	3	7	0	13
Egg	1	0	0	0	0	0	0	1
Fish	0	8	4	0	0	6	0	18
Total								32

Risk variables for the number of antibiotic residues in food animals were identified using questionnaire analysis. Risk factors for the presence of antibiotic residue contamination in fish, eggs, and chicken meat in the Yogyakarta Special Province area were evaluated using the following variables (Table-3).

**Table 3:** Observation of risk factors for antibiotic residues in food of animal origin from traditional supermarkets, and the central market in Yogyakarta Special Province.

No.	Variable	Categories	Total number	Percentage
1.	Product origin	Yogyakarta Special Province	233	77,41
		Out of Yogyakarta Special Province	68	22,59
2.	Product source	Farm	88	29,24
		Retailer shop	213	70,76
3.	Transportation mode	Motorcycle	85	28,24
		Local public transportation	5	1,66
		Private car	34	11,30
		Van	33	10,96
		Pickup	131	43,52
		Truck	13	4,32
4.	Temperature conditions when carried	Room temperature	242	80,40
		Cool	59	19,60
5.	Storage box	Cardboard	104	34,55
		Wood	72	23,92
		Plastic box	59	19,60
		Plastic shopping bag	30	9,97
		Styrofoam	30	9,97
		Tray	6	1,99
6.	Knife used	Rusty	73	24,25
		Does not rusty	228	75,75
7.	Cutting board	Wood	193	64,12
		Plastic	108	35,88
8.	Water source	Yes	182	60,47
		No	119	39,53
9.	Insect nearby	Rats	65	21,59
		Cockroach	2	0,66
		Fly	140	46,51
		Don’t know	94	31,23
10.	Sellers wear apron	Yes	137	45,51
		No	164	54,49
11.	Sellers wear gloves	Yes	9	2,99
		No	292	97,01
12.	The circumstances surrounding the seller	Dry	175	58,14
		Muddy	126	41,86
13.	Floor mats	Ceramics	185	61,46
		Cement	102	33,89
		Soil	14	4,65
Total sample			301	100

## Discussion

**Figure-3:**
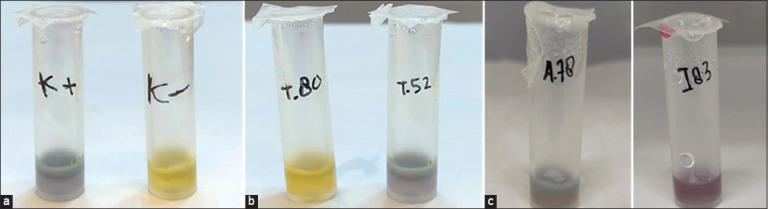
Positive results of antibiotic residue screening in egg, chicken, and fish samples. (a) The positive control is indicated by (K+), with no color change if antibiotic residue exists. A negative control Result is indicated by (K-). When there is no longer any antibiotic residue, the hue changes from purple to yellow. Positive result (T52) in the egg sample. (b) Positive result (A78) in the chicken sample. (c) Positive result (I83) in the fish sample.

A rapid test kit that inhibits the growth of the thermophilic bacterium *Geobacillus stearothermophilus* was used in the antibiotic residue screening tests on food animal samples in Yogyakarta. Positive results were observed for chicken, fish, and egg samples (Figure-3), indicating the efficacy of this test. This kit is vulnerable to antibiotics and sulfur compounds. However, the weakness of this rapid test is that it does not detect the type of antibiotics present in the sample. This rapid test will be very useful as a first step in the rapid detection of antibiotic residues in food of animal origin. For further confirmation, more accurate methods such as HPLC and gas chromatography-mass spectrometry can be tested in the laboratory.

Based on the results of the above observations, several categories can be risk factors for antibiotic residues in food of animal origin, including product source, condition of food of animal origin when transported, rusty knives, cutting boards made of wood, presence of insects around the point of sale, use of personal protective equipment by sellers, and environmental conditions around the point of sale. In the Yogyakarta Special Province region, 29.24% of the food products produced by animals sold in traditional markets, supermarkets, and central markets originate from farms. While 70.76% of food from animals comes from distributors or retailers, tracing the origin of that food product will also show that it originated from farms. It should be borne in mind that the use of antibiotics for treatment or as feed additives must be closely monitored when working on the farm. Protecting the environment, points of sale, protective clothing of sellers in markets, equipment, storage areas, water sources, insects, and floor conditions of points of sale in markets or supermarkets will therefore prevent the risk of cross-contamination between foodstuffs containing antibiotic residues and other foodstuffs that may affect antibiotic residue levels. Protecting the environment, implementing proper handling and storage practices, controlling insects, monitoring and testing food products, and raising stakeholders’ awareness can help prevent cross-contamination between antibiotic residue-containing foodstuffs and other foodstuffs [2, 17–19].

Antibiotic residues pose a risk to human health because they cause allergies, hasten the emergence of antibiotic-resistant bacteria, induce and accelerate the development of antibiotic resistance, and can lead to other serious diseases such as cancer, anaphylactic shock, nephropathy, bone marrow toxicity, mutagenic effects, and reproductive disorders [2]. The presence of antibiotic residues in food animals may also contribute to the development of antibiotic resistance [20]. The transmission of AMR during animal transport is a concern because post-journey risk factors include handling at unloading, duration of rest periods at lairage, recovery practices, re-grouping, and mixing with other animals [20]. A study revealed that wooden cutting boards used for meat processing in wet markets can harbor various pathogens, including antibiotic-resistant genes, which pose a threat to consumers. In spite of cleaning, there are pathogenic species on wooden cutting board surfaces that may contribute to the spread of diseases. Proper hygiene practices are necessary to guarantee the safety of meat sold on wooden cutting boards [21, 22].

The regulation on the use of AGPs in animal feed is an example of the ease with which antibiotics can be obtained, even if the use of certain antibiotics is no longer allowed. The highest distribution of antibiotic residues found in fish commodities in the above results is also likely due to environmental factors during maintenance, distribution, and retail. Tetracycline antibiotics can enter the body through absorption from water by injection or oral administration [23].

AMR refers to the ability of bacteria to resist the effects of antibiotics to the point that they are rendered ineffective in therapeutic settings. It can also be caused by food of animal origin [24–26]. The incidence of AMR due to the consumption of food of animal origin containing antibiotic residues may adversely affect public health and may be ineffective in clinical practice. One such adverse outcome is cancer [26–28]. A complicated and diverse problem known as AMR results when microbes become resistant to medications intended to eradicate them. Although the overuse and abuse of antibiotics in human medicine and agriculture are major contributors to AMR [29, 30], there is not much evidence to support the direct correlation between eating animal products containing antibiotic residues and cancer. Therefore, it should be noted that food originating from animals may contain antibiotic residues that may worsen antibiotic-resistant microorganisms (AMR), which is a serious threat to public health. Antibiotic residues may be found in meat and other animal products consumed by humans when antibiotics are used to promote growth or prevent illness. Humans may acquire resistant bacteria if they are regularly exposed to low doses of antibiotics in their diet [31, 32].

AMR has a significant impact on public health. This may lead to failure of treatment for bacterial infections, higher medical costs, and the emergence of resistant bacterial strains in local communities. AMR is primarily concerned with how well antibiotics treat bacterial infections, although research into possible links between AMR and other health issues, such as cancer, is currently ongoing [30, 33, 34]. Medical practitioners and regulatory organizations need to actively monitor and control the use of antibiotics in both agriculture and human health to reduce the hazards associated with AMR [26, 35]. In addition, tackling this global health issue requires establishing excellent farming practices, encouraging research on alternative strategies for animal illness prevention, and ethical use of antibiotics [36, 37].

Community education needs to address the lack of knowledge and information on the negative effects of AMR due to the consumption of food of animal origin with antibiotic residues [38, 39]. The roles of the government and important stakeholders in the food chain system are critical for guaranteeing food safety. The government and relevant stakeholders must monitor the presence of antibiotic residues in food derived from animals using reliable and accurate fast test kits such as the Premi test [18, 40]. The concept of food safety derived from hazard analysis critical control points should be applied from the farm until it is suitable for consumption to ensure food safety from food-animal origin [41, 42]. Meat, dairy products, and eggs may contain antibiotic residues because these animals use antibiotics to produce food. These residues are dangerous to the general public’s health because they can induce negative side effects and transfer bacteria that are resistant to antibiotics to people, among other health problems [43]. These residues pose a hazard to public health because they may have harmful effects and may spread antibiotic-resistant bacteria to humans. For public health and food safety, it is necessary to monitor and regulate antibiotic residues in animal feed [44]. The results of rapid antibiotic residue tests on food of animal origin in the Yogyakarta Special Province area using the Premi^®^ test kit show the presence of antibiotic residues that could jeopardize the safety of food derived from animals. Rapid test kits can be extremely helpful in facilitating the rapid and precise identification of antibiotic residues in certain food products, supporting the government’s and relevant stakeholders’ efforts to ensure food safety [45, 46]. Therefore, it is necessary to develop rapid testing kits for the detection of antibiotic residues in foodstuffs originating from animals and to use them to address this threat to public health [35, 47–50]. In conclusion, one of the most important aspects of food safety and public health is to keep an eye on antibiotic residues in animal feed. Antibiotic residues of animal origin food can effect to change the structure and function of microbiota in gut [51]. To support the government and relevant stakeholders in their efforts to guarantee the safety of food produced from animals, rapid test kits, such as Premi^®^Test (R-Biopharm AG), can significantly contribute to this. It achieves this by enabling rapid and precise detection of antibiotic residues comparing to LC-MS/MS [52]. In addition, policies should be put in place to minimize the presence of antibiotic residues in food derived from animals. Some of these methods include reducing the use of antibiotics or substituting antibiotics with alternatives.

## Conclusion

Using the rapid test kit, the presence of antibiotic residues in the food-animal origin samples from the Yogyakarta Special Province had an antibiotic residue level of 9.28% (32/345), consisting of chicken meat samples 11.3 % (13/115), fish samples 15.65% (18/115), and eggs 0.87 % (1/114). The rapid test kit used in this study is a tool that quickly assesses food safety from animal sources, including antibiotic residues, and can be used to promote socialization and education about the availability of nutritious and safe food from animal origins. However, this Premi® test kit can identify the presence of antibiotic residues in food originating from animals without providing precise levels of detection, there are certain restrictions associated with utilizing it. It is recommended that a more accurate approach, such as LC-MS/MS or HPLC, be utilized to determine antibiotic residue levels.

## Authors’ Contributions

DAW: Drafted the manuscript, designed the method, and constructed the results and discussion. RPP: Conceived and designed the study. YD and KP: Analyses and interpreted the data and reviewed the manuscript. RPP and LNF: Sampling, laboratory works, and data collections. DAW and SI: Result analysis, final review, and revised manuscript. All authors have read, reviewed, and approved the revised manuscript.
